# Correction: Optimal sizing and power losses reduction of photovoltaic systems using PSO and LCL filters

**DOI:** 10.1371/journal.pone.0304568

**Published:** 2024-05-23

**Authors:** Mohammed F. Elnaggar, Armel Duvalier Péné, André Boussaibo, Fabrice Tsegaing, Alain Foutche Tchouli, Kitmo, Fabé Idrissa Barro

The published funding statement is incorrect. The correct funding statement is as follows: This project is sponsored by Prince Sattam Bin Abdulaziz University (PSAU) as part of funding for its SDG Roadmap Research Funding Programme project number PSAU-2023-SDG-106.
