# Expression of Concern: Optimal sizing and power losses reduction of photovoltaic systems using PSO and LCL filters

**DOI:** 10.1371/journal.pone.0351344

**Published:** 2026-06-11

**Authors:** The PLOS One Editors

The *PLOS One* Editors issue this Expression of Concern for this article [1,2] because of concerns identified about the peer review process. Readers are advised to interpret the article with caution.

In addition, Reference #21 in this article was retracted before publication.
