# Supplementary material for: Optimal sizing and power losses reduction of photovoltaic systems using PSO and LCL filters
Source: PLoS One. 2024 Apr 3;19(4):e0301516. doi: 10.1371/journal.pone.0301516 (PMC10990172; doi:10.1371/journal.pone.0301516)
Supplement: S1 File — (DOCX) [file pone.0301516.s001.docx]

|  | Phase 1 |  |  | Phase 2 |  |  | Phase 3 |  |
| --- | --- | --- | --- | --- | --- | --- | --- | --- |
| 1 | 0.0505056 | 1.02329 |  | 0.0505056 | 1.02329 |  | 0.0505056 | 1.02329 |
| 2 | 0.428973 | 1.01681 |  | 0.883198 | 1.01035 |  | 1.13459 | 0.987451 |
| 3 | 1.33629 | 0.980935 |  | 1.45394 | 0.807135 |  | 2.92445 | 0.925527 |
| 4 | 2.06651 | 0.938458 |  | 2.58431 | 0.686031 |  | 3.32298 | 0.814103 |
| 5 | 2.84853 | 0.922231 |  | 3.18632 | 0.604169 |  | 4.38164 | 0.774977 |
| 6 | 3.75698 | 0.90931 |  | 4.49816 | 0.578218 |  | 5.95917 | 1.01474 |
| 7 | 4.81728 | 0.902983 |  | 5.69317 | 0.742467 |  | 7.09506 | 1.00515 |
| 8 | 5.75179 | 0.906467 |  | 6.01033 | 1.02787 |  | 8.53431 | 1.00219 |
| 9 | 6.21235 | 1.02792 |  | 7.52452 | 1.00853 |  | 9.38787 | 0.900702 |
| 10 | 7.42351 | 1.0085 |  | 8.76191 | 1.0088 |  | 10.1761 | 1.00911 |
| 11 | 8.61072 | 1.01532 |  | 9.94343 | 0.900824 |  | 10.7764 | 0.894446 |
| 12 | 9.84421 | 0.936879 |  | 10.7292 | 0.960031 |  | 11.5633 | 0.976612 |
| 13 | 10.8814 | 0.973183 |  | 11.7145 | 0.970086 |  | 12.5725 | 0.957154 |
| 14 | 12.472 | 0.966971 |  | 12.6675 | 0.835824 |  | 13.2229 | 0.832666 |
|  |  |  |  |  |  |  |  |  |

**Supporting Information files**

1. **Extracted points using PSO algorithms in MATLAB on IEEE 14 Bus**
2. **PV side parameters**

| **Input and ouput parameters** | **Values** |
| --- | --- |
| Input Voltage value | 100 V |
| Output Voltage value | 805 V |
| Inductors | 301 × 10^−6^ H |
| Inductor (*L*_3_) | 200 × 10^−6^ H |
| Capacitors values | 3.5 × 10^−6^ F |
| DC Capacitor | 520 × 10^−6^ F |
| Duty Cycle | 85% |
| Switching Frequency | 55 kHz |
| Load value | 253 Ω |

**3. Grid side parameters**

| **Parameters** | **Set Values** |
| --- | --- |
| Grid nominal Voltage | 220 V rms |
| Grid frequency | 50 Hz |
| Converter input inductor (*L_i_*) | 1.8 × 10^−6^ H |
| Converter resistance value | 0.25 ohm |
| Grid inductor value | 2.32 × 10^−4^ H |
| Grid resistance value | 0.33 ohm |
| LCL value | 4.55 × 10^−6^ F |
| LCL damping resistance value | 33.6 ohm |
| DC bus voltage | 600 V |
| Grid resistance value | 3.2 ohm |
| Grid inductance value | 4.2 × 10^−4^ H |
| Switching frequency value | 21.5 × 10^−4^ Hz |
